# GSTA1 diplotypes affect busulfan clearance and toxicity in children undergoing allogeneic hematopoietic stem cell transplantation: a multicenter study

**DOI:** 10.18632/oncotarget.20310

**Published:** 2017-08-27

**Authors:** Marc Ansari, Patricia Huezo-Diaz Curtis, Chakradhara Rao S. Uppugunduri, Mohammed Aziz Rezgui, Tiago Nava, Vid Mlakar, Laurence Lesne, Yves Théoret, Yves Chalandon, Lee L. Dupuis, Tao Schechter, Imke H. Bartelink, Jaap J. Boelens, Robbert Bredius, Jean-Hugues Dalle, Saba Azarnoush, Petr Sedlacek, Victor Lewis, Martin Champagne, Christina Peters, Henrique Bittencourt, Maja Krajinovic

**Affiliations:** ^1^ Department of Pediatrics, CANSEARCH Research Laboratory, Faculty of Medicine, Geneva, Switzerland; ^2^ Department of Pediatrics, Onco-Hematology Unit, Geneva University Hospital, Geneva, Switzerland; ^3^ Charles-Bruneau Cancer Center, CHU Sainte-Justine Research Center, Montreal, Quebec, Canada; ^4^ Department of Pharmacology, Faculty of Medicine, University of Montreal, Montreal, Quebec, Canada; ^5^ Clinical Pharmacology Unit, CHU Sainte-Justine, Montreal, Quebec, Canada; ^6^ Faculty of Medicine, Federal University of Rio Grande do Sul, Porto Alegre, Brazil; ^7^ Department of Medical Specialties, Division of Hematology, Geneva University Hospital, Geneva, Switzerland; ^8^ Department of Haematology/Oncology, Blood and Marrow Transplant Unit, The Hospital for Sick Children, Toronto, Ontario, Canada; ^9^ Pediatric Blood and Marrow Transplantation Program, University Medical Center, Utrecht, The Netherlands; ^10^ Department of Pediatrics, Center of Infectious Diseases, Leiden University Medical Center, Leiden, The Netherlands; ^11^ Pediatric Hematology Department, Robert Debré Hospital, Assistance Publique, Hôpitaux de Paris, Paris, France; ^12^ Department of Pediatric Hematology and Oncology Teaching Hospital, 2nd Medical School, Charles University, Prague, Czech Republic; ^13^ Department of Pediatrics, Alberta Children’s Hospital, Calgary, Alberta, Canada; ^14^ Department of Hematology, Hospital Verdun, Montreal, Quebec, Canada; ^15^ Department of Pediatrics, Stem Cell Transplantation Unit, St Anna Children’s Hospital, Vienna, Austria; ^16^ Department of Pediatrics, Faculty of Medicine, University of Montreal, Montreal, Quebec, Canada; ^17^ Department of Medicine, The University of California San Francisco, San Francisco, CA, USA; ^18^ On Behalf of the Pediatric Disease Working Party of the European Society for Blood and Marrow Transplantation, Leiden, The Netherlands

**Keywords:** busulfan, pharmacokinetics, pharmacogenetics, toxicity, hematopoietic stem cell transplantion

## Abstract

Busulfan (BU) dose adjustment following therapeutic drug monitoring contributes to better outcome of hematopoietic stem cell transplantation (HSCT). Further improvement could be achieved through genotype-guided BU dose adjustments. To investigate this aspect, polymorphism within glutathione S transferase genes were assessed. Particularly, promoter haplotypes of the glutathione S transferase A1 (*GSTA1*) were evaluated *in vitro,* with reporter gene assays and clinically, in a pediatric multi-center study (N =138) through association with BU pharmacokinetics (PK) and clinical outcomes. Promoter activity significantly differed between the *GSTA1* haplotypes (p<0.001) supporting their importance in capturing PK variability. Four *GSTA1* diplotype groups that significantly correlated with clearance (p=0.009) were distinguished. Diplotypes underlying fast and slow metabolizing capacity showed higher and lower BU clearance (ml/min/kg), respectively. *GSTA1* diplotypes with slow metabolizing capacity were associated with higher incidence of sinusoidal obstruction syndrome, acute graft versus host disease and combined treatment-related toxicity (p<0.0005). Among other *GST* genes investigated, *GSTP1 313GG* correlated with acute graft versus host disease grade 1-4 (p=0.01) and *GSTM1 non-null* genotype was associated with hemorrhagic cystitis (p=0.003). This study further strengthens the hypothesis that *GST* diplotypes/genotypes could be incorporated into already existing population pharmacokinetic models for improving first BU dose prediction and HSCT outcomes. (N^o^ Clinicaltrials.gov identifier: NCT01257854. Registered 8 December 2010, retrospectively registered).

## INTRODUCTION

Myeloablative conditioning regimens comprising the bi-functional alkylating agent busulfan (BU) were introduced in the late 1970s as an alternative to total body irradiation [1]. Since then BU has been extensively used, especially in combination with cyclophosphamide in patients undergoing hematopoietic stem cell transplantation (HSCT) [2]. Studies in children and adults demonstrate reduced toxicity and increased efficacy, when the BU area under the curve concentration (AUC) is within optimal target range [3, 4]. Hence, dose adjustment guided by therapeutic drug monitoring is performed to prevent treatment-related toxicities. In a recent study comparing the performance of 12 pediatric dosing guidelines, the therapeutic target window can be reached in approximately 51% to 74% in pediatric cases and 45% to 64% in infants [5]. Nevertheless, the authors and others also caution about emerging evidence that the therapeutic AUC range of 3.7 - 6.2 mg.h/L per dose (equivalent ∼ steady state concentration, Css, of 615 - 1031.3 ng/mL) should not be universally applied as the optimal AUC may depend on the indication for transplant or other patient-related factors [5-8]. Recently, a narrower optimal i.v. BU cumulative AUC of 78-101 mg.h/L (equivalent to a Css of ∼ 830-1050 ng/mL) has been suggested to improve outcomes irrespective of disease condition, based on the analysis of 674 pediatric patients [4]. This proposed narrower therapeutic window constitutes a need to identify the underlying factors responsible for the inter-individual variability of BU clearance (CL), particularly at first dose before BU adjustment.

BU is eliminated via conjugation with glutathione catalyzed by glutathione S-transferase enzymes (GST) especially by the Glutathione S Transferase Alpha1 (GSTA1) isoform followed, to a lesser extent, by the Glutathione S Transferase Mu 1 (GSTM1) and Glutathione S Transferase Pi 1 (GSTP1) [9]. Factors that could affect the metabolism or elimination of BU are the availability of glutathione, efflux of conjugates by transporter proteins Unmarked set by huezodia and the different activity of GSTs [2, 10]. The GSTs are highly polymorphic; the promoter region of *GSTA1* contains polymorphic variants that influence enzyme function [3, 11, 12]. A null variant is encountered for *GSTM1*, whereby the entire gene is deleted in a considerable proportion of different populations, resulting in the complete absence of the corresponding enzyme activity [13]. *GSTP1* contains the *313A>G* polymorphism leading to an Ile-to-Val substitution that has shown to decrease enzyme activity [14]. Our team and other groups have investigated genetic variants in *GSTs* for their association with BU exposure and/or clinical outcomes [15-33], summarized in Supplementary Table 1. Most studies demonstrated an association between BU pharmacokinetics (PK) and *GSTA1*-69 C>T, [15-28] which delineates haplotypes *A and *B. Nevertheless, functional assessment of *GSTA1* sub-haplotypes and more detailed insight into their relationship with clinical outcomes in a larger patient population is still lacking. In this report, we analyzed promoter activity of each *GSTA1* haplotype subgroup and have extended our previous analysis of pediatric patients from a single center [20] to a larger multicenter cohort to validate the association of *GST* genes, particularly *GSTA1* haplotype combinations as diplotypes, with BU exposure and clinical outcomes of HSCT.

## RESULTS

### Functional characterization of the *GSTA1* polymorphisms

**Figure 1:**
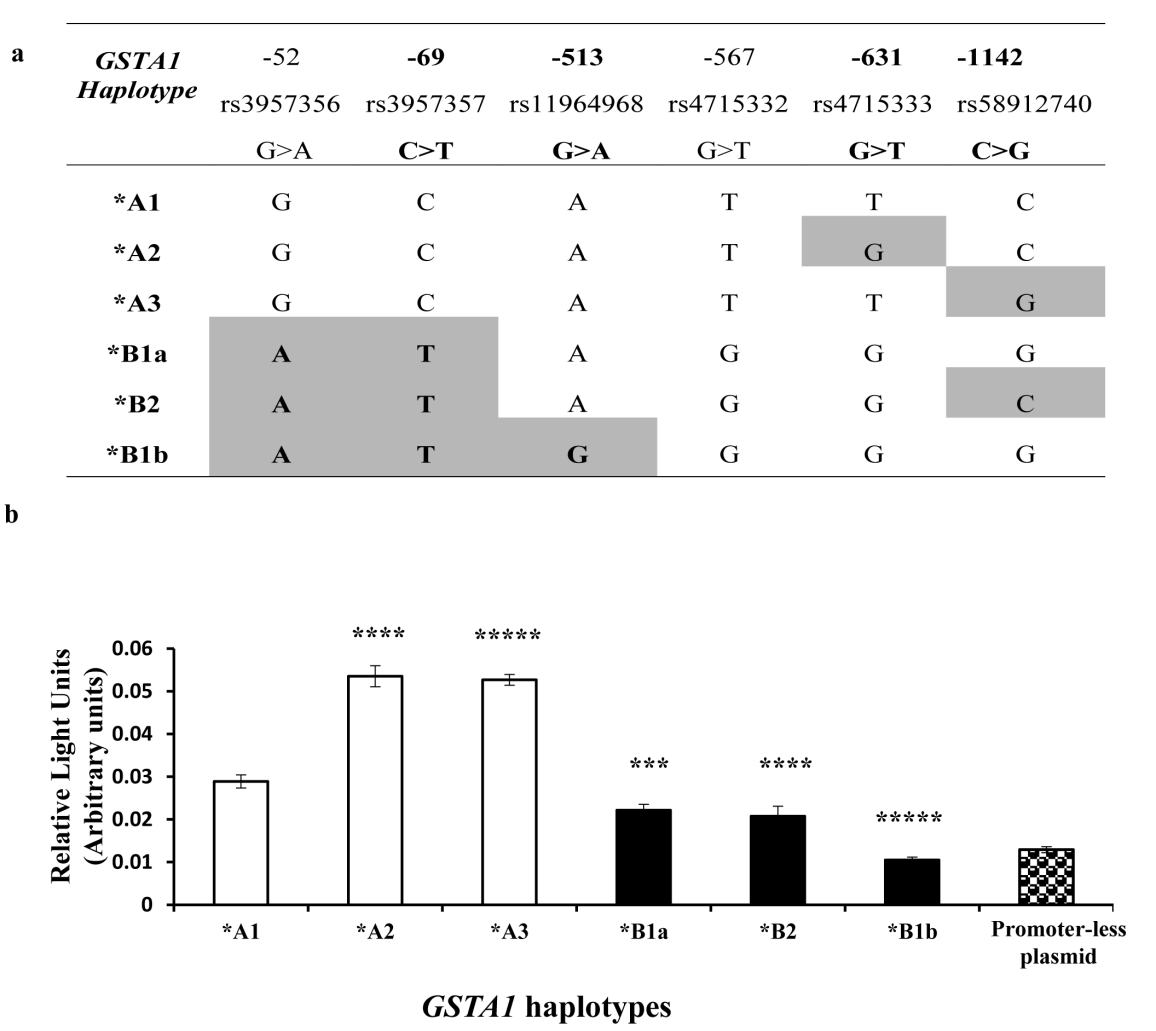
*GSTA1* Haplotype and Reporter Gene assay of *GSTA1* promoter **A.** Haplotypes investigated with luciferase reporter assay. rs (reference SNP ID) numbers correspond to each SNP included for site directed mutagenesis. SNPs used for genotyping and for inferring sub-haplotypes in patients are highlighted in bold. **B.** Luciferase activities of the proximal promoters of *GSTA1* variants (GSTA1*A1, GSTA1*A2, GSTA1*A3, GSTA1*B1a, GSTA1*B2, GSTA1*B1b) in transient transfection in HepG2 cells. Error bars represent the standard deviations. Pairwise comparisons by analysis of variance (ANOVA) between data for the GSTA1*A1 vs. any other haplotype, after Bonferroni correction *** = *p* < 0.001; ****= *p* <0.0001, ***** = *p* < 0.000001.

To explore how *GSTA1* function is related to each *GSTA1* haplotype (Figure 1a), we estimated promoter activity by luciferase gene reporter using six haplotype constructs that were transiently transfected in human hepatoma (HEPG2) cells. Results are illustrated in Figure 1b, where a significant increase of luciferase activity was observed when *A1 was mutated at position -631 forming *A2 and at position -1142 forming*A3 haplotype (*p* < 0.001). In contrast, the promoter activity was significantly decreased in the case of all *B haplotypes that are conjunctly delineated from haplotype *A by changes at three positions in full linkage disequilibrium (-52, -69, -567). The lowest activity among *B haplotypes was observed for *B1b (defined by position -513, *p* = 0.00001) that equalled the activity of the promoterless plasmid.

### Pharmacogenetics vs pharmacokinetics and dose requirement

**Figure 2:**
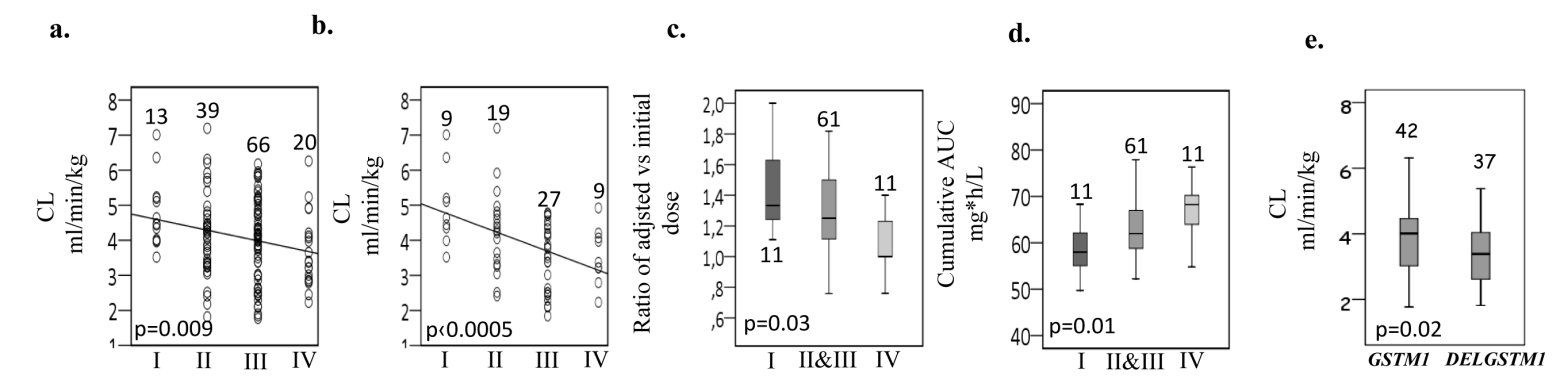
Pharmacokinetic parameters of BU and dose requirement in relation to *GSTA1* functional diplotype groups and *GSTM1* genotypes **A.** Busulfan first dose clearance (CL, in ml/min/kg) against *GSTA1* diplotypes **B.** Busulfan first dose CL in females only against *GSTA1* genotypes. **C.** Dose requirement (ratio of adjusted to initial dose) against *GSTA1* diplotypes. **D.** Cumulative AUC (mg.h/L) against *GSTA1* diplotypes. CHU Sainte-Justine patients only were included for analysis presented in C and D. Diplotype *groups II* and *III* were combined into a single group in C and D. **E.** Busulfan first dose clearance in children above 4 yrs of age against *GSTM1* genotypes. DELGSTM = Deleted *GSTM1* gene. Number of patients and p values are depicted on the plots.

Based on the functional effect of each haplotype, predicted activity of each diplotype and the relationship with CL (ml.min/kg), four major functional *GSTA1* groups were revealed Table 1; I (in 9.4% cases), defined by two copies of rapid metabolizing alleles, mostly represented by *A2*A2 individuals, *group IV* (14.5%) represented by two copies of slow metabolizing alleles (defined in all cases but one by*B1a*B1a diplotype) and by the presence of one copy of very slow metabolizing *B1b allele. *Group II* (28.2%) and *III* (47.8%) had intermediate to normal metabolizing capacity and were defined by the presence of *A2 and *A1, respectively. Linear effect was observed, whereby *group I* demonstrated highest and *group IV* lowest CL (*p* = 0.009, Figure 2a) with even more evident correlation seen in girls (*p* ≤ 0.0005, Figure 2b).

**Table 1:** *GSTA1* diplotype frequencies in the study population and proposed functional groups

*GSTA1* Diplotype	Diplotype Frequencies N (%)	Proposed Functional group
GSTA1*A2*A2	12 (8.7)	I (9.4%)
GSTA1*A2*A3	1 (0.7)	
GSTA1*A2*A1	18 (13.0)	II (28.2%)
GSTA1*A2*B1a	11 (8.0)	
GSTA1*A2*B2	10 (7.2)	
GSTA1*A1*A1	17 (12.3)	III (47.8%)
GSTA1*A1*B1a	48 (34.8)	
GSTA1*A1*B2	1 (0.7)	
GSTA1*B1b*A2	3 (2.2)	IV (14.5%)
GSTA1*B1a*B1a	10 (7.2)	
GSTA1*B2*B1a	1 (0.7)	
GSTA1*B1b*A1	3 (2.2)	
GSTA1*B1a*B1b	3 (2.2)	
***Total***	138 (100)	

Abbreviations: *GSTA1*: glutathione S transferase alpha1. The proposed functional groups arise from in vitro reporter-gene assays and in vivo PK data performed in this study. Group I: considered as rapid metabolizers; Group II: considered as intermediate metabolizers; Group III: considered as normal metabolizers and Group IV: considered as slow metabolizers.

Due to the difference in dose adjustment across participating centers, the ratio of adjusted to initial dose and cumulative AUC obtained in a single center (University Hospital Center Sainte Justine, Montreal) was compared among GSTA1 diplotype groups. Patients in *group I* had on average a higher dose requirement compared to the other patients, whereas *group IV* cases had on average very little change from the initial dose (*p* = 0.03, Figure 2c). Cumulative AUC was also significantly associated in an additive manner with four diplotype groups with the highest exposure seen in the *group IV* (*p* = 0.01, Figure 2d).

Genotype frequencies for *GSTM1* and *GSTP1* variants are summarized in Table 2. When all patients were analyzed there was no significant association between PK and *GSTP1* or *GSTM1* genotypes (Table 3). But BU CL was associated with *GSTM1* genotypes in children above 4 years of age (Figure 2e). *GSTA1* diplotypes correlated with different ethnicities with *group I* being more frequent in other populations than in Caucasians (*p* < 0.0005). Among non-genetic factors, CL adjusted for weight correlated significantly with age (*p* < 0.0005). Age (*p* < 0.0005), gender (*p* = 0.09) and *GSTA1* diplotypes (*p* = 0.01) were retained in the final multivariate linear model that explained 28% of overall variability in BU CL (Table 3). The diplotype contribution was relatively minor (5%) when all diplotypes and all patients are included in the analysis. However, when all girls (*n* = 64) or patients with diplotype *groups I* and *IV* representing the extreme of CL distribution (*n* = 33) were considered, diplotype contribution increased to 19% and 23%, respectively, and the model explained 46% and 42% of variability in CL.

**Table 2:** *GSTM1* and *GSTP1* genotypes and minor allele frequencies in the study population

Genetic variant	Homozygous for major allele N (%)	Heterozygous N (%)
*GSTM1null* *	73 (52.9)	-
***GSTP1 313A>G***	52 (37.7)	69 (50)
***GSTP1 341C>T***	117 (84.8)	18 (13)

Abbreviations: *GSTM1*: glutathione S transferase Mu1; *GSTM1* null- homozygous individuals for deletion; *GSTP1*: glutathione S transferase Pi1. *Distinction cannot be made between *GSTM1* non-null heterozygous and homozygous individuals by the method used in the study, therefore observed frequencies could not be provided. *GSTM1* null individuals are considered as homozygous for major allele.

**Table 3:** Relationship between GST genotypes and the clearance in univariate and multivariate linear regression

	Variables	*p*	B	R^2^	Model
*All patients*	*GSTA1*	*0.009*	-0.31	5	Univariate
	*GSTA1*	*0.01*	-0.27	28	Multivariate
	Age	*<0.0005*	-0.09		
	Sex	*0.09*	0.3		
Girls only	*GSTA1*	*<0.0005*	-0.54	19	Univariate
	*GSTA1*	*0.006*	-0.35	46	Multivariate
	*Age*	*<0.0005*	-0.1		
Group I and IV	*GSTA1*	*0.005*	-0.37	23	Univariate
	*GSTA1*	*0.02*	-0.28	42	Multivariate
	*Age*	*0.004*	-0.1		

Co-variables include age (continuous), sex (dichotomized), conditioning regimen (busulfan-cyclophophamide vs others) and diagnosis (malignant versus non-malignant); Co-variables with p<0.1 were retained in final model. When all patients and girls only were analyzed, *GSTA1* included all 4 diplotype groups; B, unstandardized coefficient, R^2^, % of variability explained by the genotype or the model (in multivariate analysis).

### Pharmacogenetics vs HSCT-related toxicities

**Figure 3:**
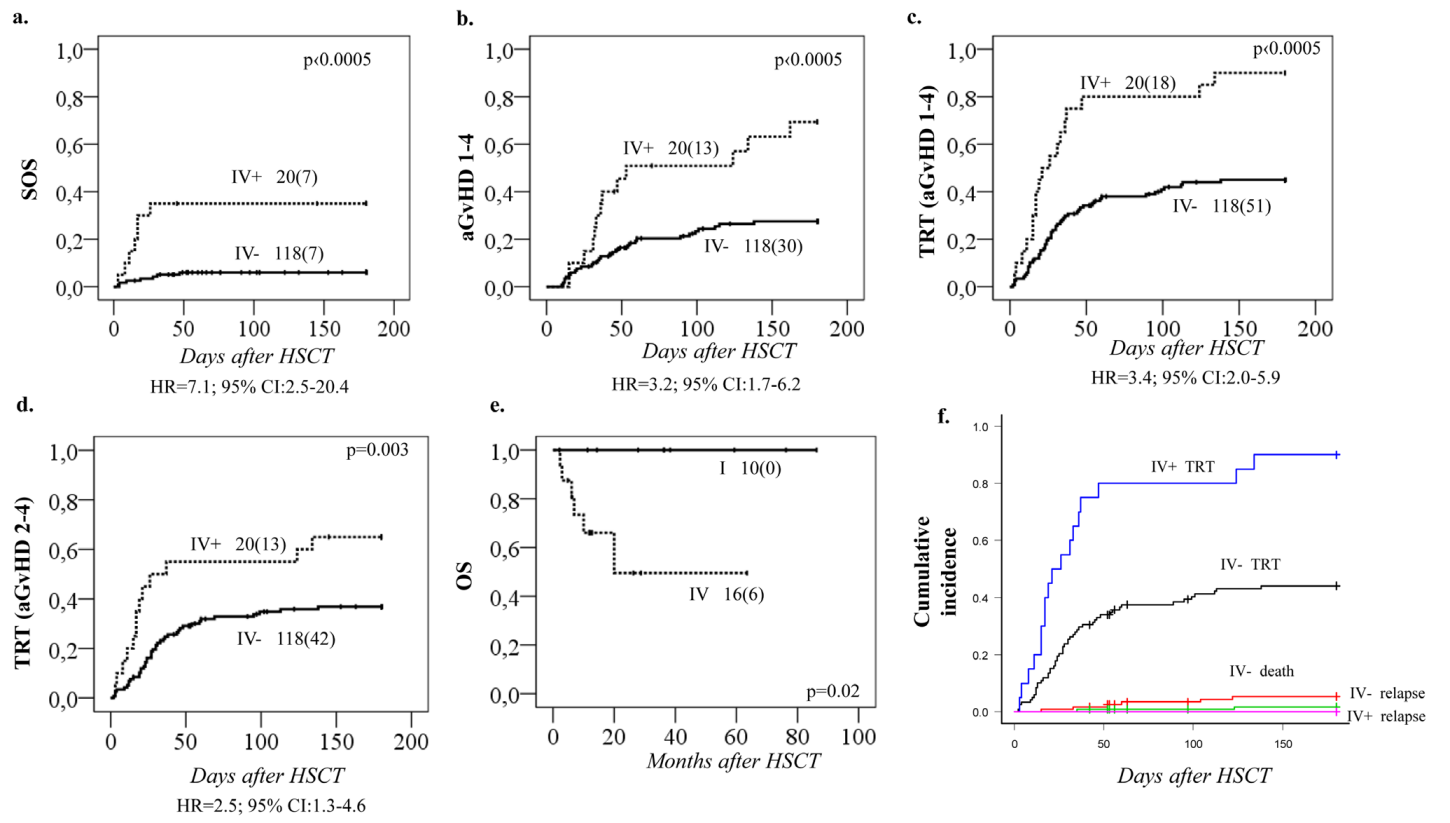
Incidence of SOS, aGvHD and TRT in relation to *GSTA1* functional diplotype groups Cumulative incidences of **A.** sinusoidal obstruction syndrome (SOS), **B.** acute graft versus host disease (aGvHD) 1-4, **C.** treatment related toxicity (TRT) including aGvHD 1-4 and **D.** TRT including aGvHD 2-4. Results plotted for diplotype *group IV (IV+)* versus *groups I, II & III (IV-)*. **E.** Overall survival (OS) in relation to *GSTA1* extreme diplotype status (*group I vs. group IV*), in patients who received busulfan-cyclophosphamide conditioning regimen. Total number of patients represented by each curve with number of patients with indicated toxicities in parenthesis, and p value are depicted on each plot; *group IV* associated hazard ratios are depicted below each plot. **F.** Association of TRT with diplotype group IV in a competing events risk analysis. IV+ and IV- indicates the presence of this *GSTA1* diplotype group. Competing events for TRT incidence were: death and relapse. p values for the difference in cumulative incidence of TRT, death, and relapse, between haplotype groups (IV vs others) is 0.000003, 0.3 and 0.5, respectively.

Analyses between HSCT-related toxicities and the four *GSTA1* diplotype groups revealed a strong association with Sinusoidal Obstruction Syndrome (SOS) (*p* < 0.0005; Figure 3a), whereby *Group IV* carriers had seven-fold higher risk of SOS (HR = 7.1; 95% CI: 2.5-20.4) compared to patients with other *GSTA1* diplotypes. Likewise, *group IV* carriers were also associated with the highest risk of acute Graft versus Host Disease (aGvHD), grades 1-4 (*p* < 0.0005, Figure 3b) and with Treatment Related Toxicity (TRT: combining SOS, hemorrhagic cystitis, lung toxicity and aGvHD grades 1-4, *p* < 0.0005, Figure 3c). The association between *GSTA1* diplotypes and TRT was also maintained if aGvHD grades 2-4 were included in the analysis rather than all grades of aGvHD (*p* = 0.03, Figure 3d). Individuals with *group IV* who received BU-cyclophosphamide conditioning regimen had also lowest overall survival (OS) compared to *group I* (*p* = 0.02, Figure 3e). This was likely due to the high TRT rates, since when the cumulative incidence of competing events analysis was performed, with TRT, relapse and death as competing events, only the association with TRT remained significant (*p* < 0.0005, Figure 3f).

**Figure 4:**
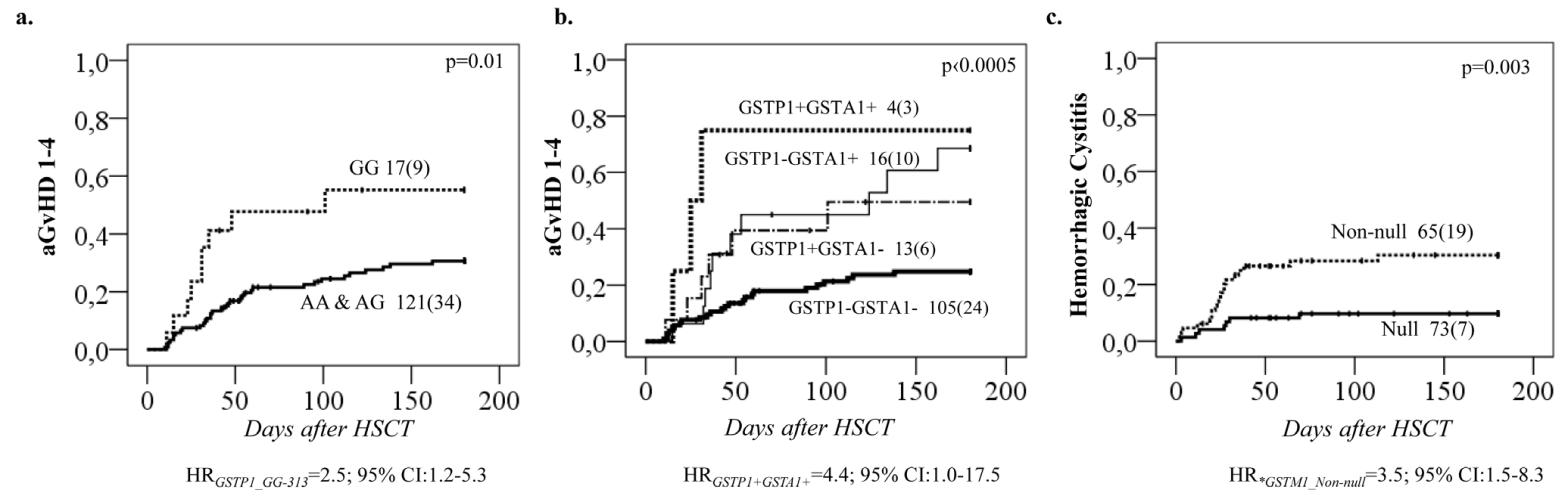
Complications of HSCT in relation to *GSTP1* genotypes and *GSTA1* diplotypes **A.** Acute GvHD 1-4 incidence according to *GSTP1* c.313A>G genotypes; and **B.** Acute GvHD 1-4 incidence according to combinatory *GSTA1-GSTP1* status. A plus sign represents the risk genotypes, which is presence of GSTP1*GG and/or *GSTA1* diplotype *group IV*. **C.** Hemorrhagic Cystitis (HC) incidences in relation to *GSTM1 Null* and *Non-null* genotype.

**Figure 5:**
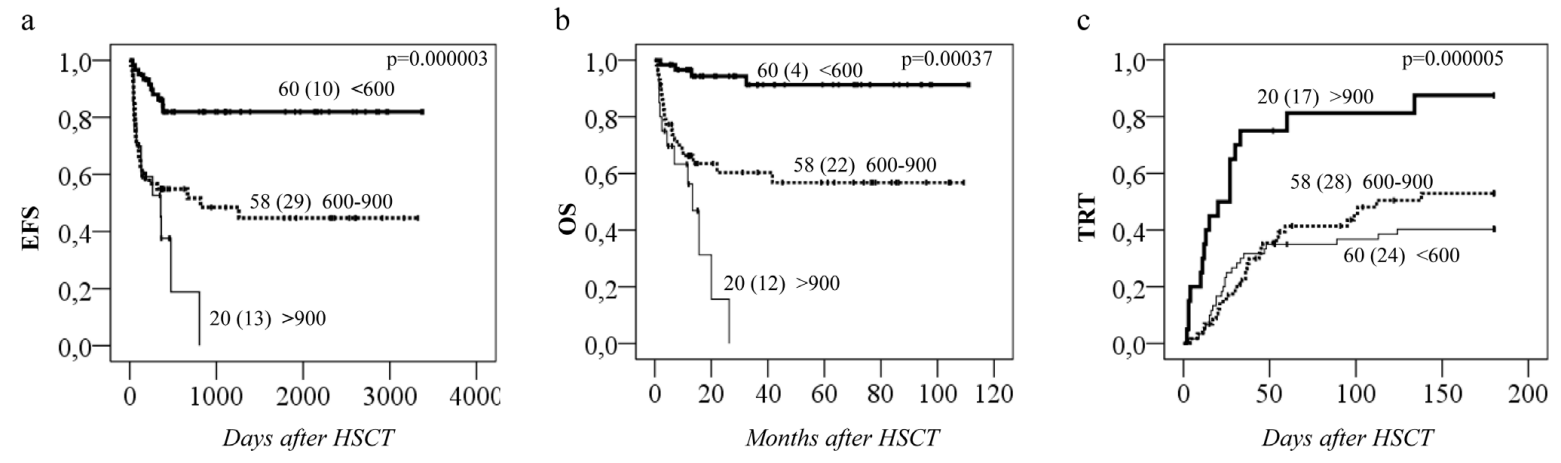
Busulfan plasma exposure and clinical outcomes of HSCT Incidences of event-free survival (EFS), overall survival (OS), and treatment related toxicity (TRT) plotted against 3 groups based on first dose steady state concentration (Css) i.e. <600, 600-900 and > 900 ng/mL in all patients (*n* = 138). Total number of patients in each group (number of patients with events) are depicted on all plots. *P* values are shown on the plots.

**Figure 6:**
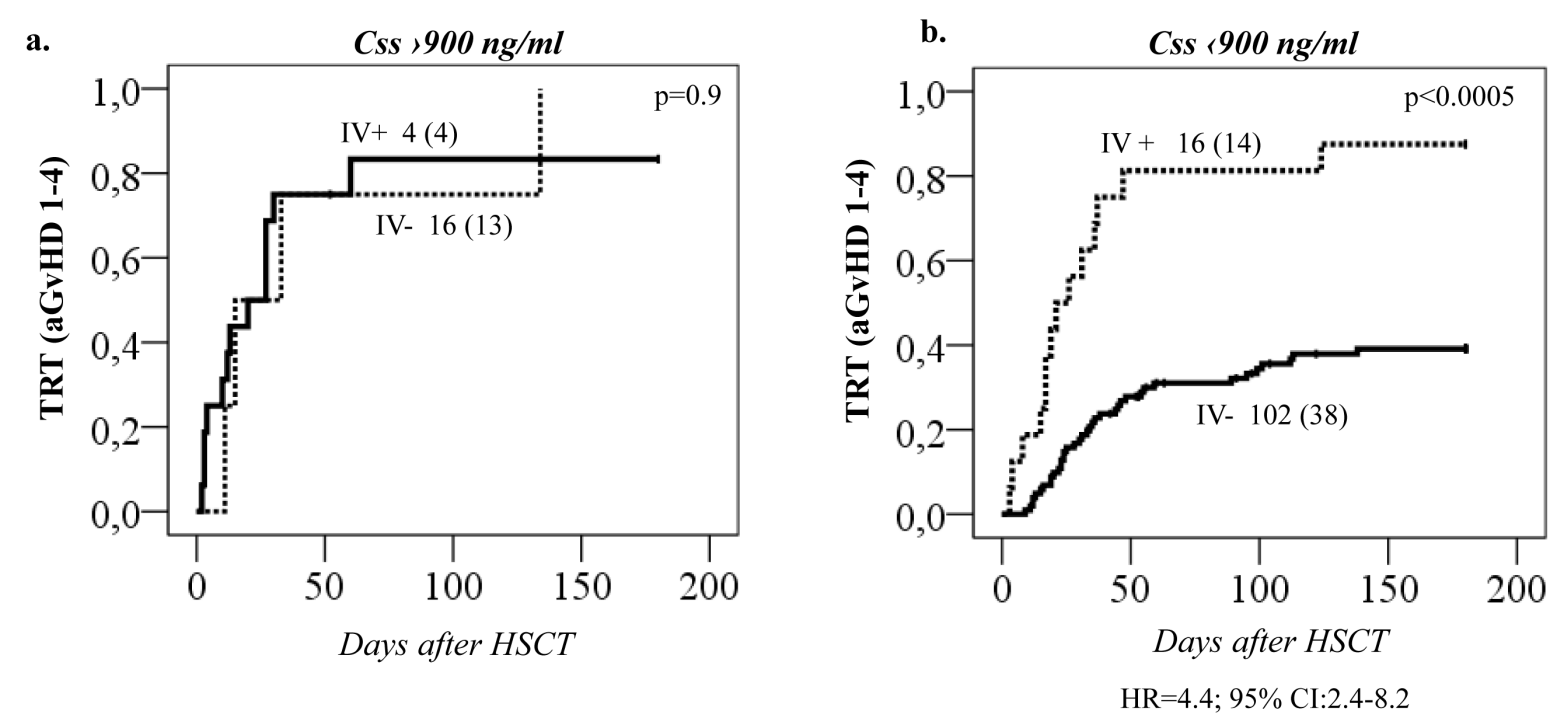
Treatment related toxicity (TRT) in relation to both 1st dose Css and *GSTA1* groups TRT (all cases combined) plotted against **A.** Busulfan Css below 900 ng/mL or **B.** Css above 900 ng/mL, dependent on whether patients are in *GSTA1* diplotype *group I, II, III (*IV-*)* or *IV (*IV+*)*. Total number of patients in each group with number of patients with TRT in brackets is depicted on all plots. Hazard ratio for group IV is depicted for plot (A) only.

Regarding the remaining *GST* genes, *GSTP1* (*GG 313*) was associated with a higher probability of aGvHD 1-4 (*p* = 0.01, Figure 4a). This effect was independent of *GSTA1* haplotype and each genotype (*GSTP1 GG 313* and *GSTA1 group IV*) independently and combined contributed to aGvHD development with highest risk seen in individuals with both risk genotypes (*p* < 0.0005, Figure 4b). Incidence of hemorrhagic cystitis was higher in *GSTM1 non-null* individuals compared to patients with *GSTM1* deletion (*p* = 0.003, Figure 4c). Css after first dose categorized according to historical target correlated with event free survival (EFS), OS and TRT (*p* < 0.0005) (Figure 5a). Css above 900 ng/mL was associated with TRT irrespective of diplotype groups (Figure 6a), whereas high risk of TRT for Css below 900 ng/mL was evident only for *group IV* carriers (Figure 6b). In multiple logistic regression models the best predictors of SOS and TRT were age and *GSTA1* diplotypes (Table 4a), explaining 21-22% of variability of which 15-17% was attributed to *GSTA1*. For aGvHD, the final model included conditioning regimen, *GSTA1* and *GSTP1* that explained 19% of the variability of which 14% was accounted for by the two GSTs (Table 4a). Multivariate modelling for hemorrhagic cystitis included *GSTM1*, diagnosis, conditioning regimen and age, explaining 47% of variability of which 10% was attributed to *GSTM1*(Table 4a). BU exposure was significantly associated with aGvHD, TRT and hemorrhagic cystitis and was further analyzed together with genotypes/diplotypes in a multivariate model in which both variables remained significant predictors of respective outcomes (*GSTA1*, *p* = 0.003, *GSTM1*, *p* = 0.005, Css *p* ≤ 0.05, (Table 4b).

**Table 4:** Relationship between GST genotypes and other variables with clinical outcomes in univariate and multivariate logistic regression

	Clinical outcome	Variables	p	OR (95% CI)	R^2^	Model
**A**	**SOS**	*GSTA1*	<0.0005	8.5 (2.6-28.2)	16.7	Univariate
		*GSTA1*	0.001	9.0 (2.6-31)	21	Multivariate
		Age	0.07	1.1 (1.0-1.2)		
	**aGvHD** **1-4**	*GSTA1*	0.001	5.2 (1.9-14.5)	14.1	Multivariate with genotypes only
		*GSTP1*	0.08	2.7 (0.9-7.9)		
		*GSTA1*	0.001	6.0 (2.1-17.4)	19.4	Multivariate
		*GSTP1*	0.03	3.6 (1.1-11.4)		
		Conditioning	0.02	0.3 (0.1-0.8)		
	**TRT**	*GSTA1*	0.001	11.8 (2.6-53.3)	15.4	Univariate
		*GSTA1*	0.001	12.7 (2.8-58.2)	22.3	Multivariate
		Age	0.008	1.1 (1.0-1.2)		
	**HC**	*GSTM1*	0.005	3.9 (1.5-10.0)	10.1	Univariate
		*GSTM1*	0.002	6.6 (2.0-22.2)	47.1	Multivariate
		Age	<0.0005	1.3 (1.1-1.4)		
		Conditioning	0.06	9.3 (0.9-96.4)		
		Diagnosis	0.1	2.8 (0.8-10.2)		
						
**B**	**Clinical outcome**	**Variables**	**p**			
	**aGvHD** **1-4**	*GSTA1*	0.003	4.8 (1.7-13.5)	17.5	Multivariate
		*GSTP1*	0.06	2.8 (0.9-8.6)		
		Css	0.05	1.0 (1-1)		
	**TRT**	*GSTA1*	0.003	10.4 (2.3-47.8)	23.8	Multivariate
		Css	0.003	1 (1-1)		
	**HC**	*GSTM1*	0.005	4.1 (1.5-10.9)	19.1	Multivariate
		Css	0.004	1 (1-1)		

In A: co-variables include age, sex, diagnosis and conditioning regimen; in B: co-variable is Busulfan exposure represented as Busulfan Steady state concentration (Css). Co-variables with p<0.1 were retained in final model. Dichotomized variables were *GSTP1* (GG vs others), *GSTA1* (group IV vs others), *GSTM1* (non-null vs null), conditioning regimen (busulfan-cyclophosphamide vs others) and diagnosis (malignant versus non-malignant) whereas Css and age were continuous variables, OR, odds ratio, CI, confidence interval, R^2^, % of variability explained by the genotype or the model (in multivariate analysis).

## DISCUSSION

This study is conducted in a multicenter pediatric HSCT cohort and provides the first evidence for the association of functional *GSTA1* diplotype groups with BU PK and clinical outcomes. Several studies including our own (summarized in Supplementary Table 1), performed in childhood and adult patients who received either iv or oral BU, have previously demonstrated an association between first dose PK and *GSTA1* gene [3, 15-20, 22-26, 28, 33]. These studies, however, rarely included clinical outcome and none (except the one conducted by our group) included *GSTA1* sub-haplotypes. There are only two studies [19, 26] that are comparable to the present report since they were conducted in childhood patients diagnosed mainly with malignancies who received i.v. BU- cyclophosphamide combination. Still, genetic association with clinical outcomes were not investigated in both of these studies and were also limited by the sample numbers. Overall, positive associations between *GSTA1* gene and PK are characterized by higher BU exposure in *B individuals and lower in *A carriers, which is functionally driven by a -52 G>A promoter SNP (in complete linkage disequilibrium with -69 C>T and -567 A>T) delineating *A and*B haplotypes [34]. *GSTA1* gene has an additional three SNPs within its promoter further diversifying haplotypes into subgroups that can potentially contribute to *GSTA1* functionality. Using a gene reporter assay, we defined for the first time the promoter activity of each subgroup showing that *A1 could be classified as a normal metabolizer, *A2 and *A3 as rapid metabolizers, whereas slow metabolizing capacity was assigned to *B1a and *B2 and very slow to *B1b. Four major diplotype groups that reflected well *GSTA1*-PK relationship were defined. *Group I* (rapid metabolizers) was mostly represented by *A2*A2 diplotype and group IV (slow metabolizers) by *B1a*B1a diplotype and the presence of *B1b haplotype. *Group I* correlated with highest and *group IV*, with lowest CL. The relationship observed with *group I* is in accordance with our previous study [20, 33] which reported higher BU CL in*A2 *A2 individuals. More evident association of diplotype groups with PK was seen in girls, also in accordance with previous observation [20], which could be due to the higher level of *GSTA1* in females compared to males particularly in liver cytosol [35, 36], or to more prominent induction and inhibition of *GSTA1* [37].

Association of *GSTA1* with PK was also reflected in *GSTA1* association with dose requirements and with cumulative AUC, as seen by the analysis performed in CHU Sainte Justine patients. Indeed, on average there was no dose reduction in *group IV*, whereas in *group I*, in spite of the dose increase in most of these patients, cumulative AUC stayed the lowest. For *group IV* higher cumulative AUC’s may suggest that 1) Initial doses were too high for some patients; 2) Subsequent dose adjustments in these patients were not sufficient and thus dose reduction needs to be greater; 3) Toxic damage may have already been inflicted after first doses, 4) Possibility of a significant reduction in CL in *group IV* upon time. This emphasizes the need to adjust first dose according to genotype rather than adjusting only after therapeutic drug monitoring. Our results are in accordance with a study looking at oral BU dose requirement based on *GSTA1* haplotypes [18] in which the first two oral doses were kept constant and later doses were adjusted to target a Css of 900 ng/mL (AUC of 5.4 mg.h/L), but despite dose adjustments, the average BU Css of the 3 dosing intervals remained significantly higher among GSTA1*B carriers [18].

We did not observe an association of *GSTM1* with PK when all patients were analyzed. However, *GSTM1*’s involvement in BU PK could be quantitatively and qualitatively different in infants and toddlers (0-4 years old) compared to children and adolescents (4-18 year olds) as a consequence of developmental changes in gene expression as reported in ontogeny data [38]. This shows the limitation of the model with CL in L/h/kg that does not capture well the data for all patients over a varied age range. In future studies a more physiological related PK model should be used [39, 40]. Indeed, similar to our previous finding [20] patients above 4 years of age with *GSTM1* null genotype had lower CL (Figure 2e). Bremer and colleagues [18] reported that GSTA1*B*B individuals who are also *GSTM1* null tend to have higher Css after oral BU and patients with a combined GSTA1*B*B, *GSTM1* null and *GSTT1* null presented the highest Css levels of all genotype groups. We did not notice the combined effect of *GSTA1* diplotypes and GSTM1 null genotype on the PK. Oral BU used in the study of Bremer et al., [18] usually resulting in larger inter-individual variability, might have also contributed to this difference.

Attention should be given to the association of *GSTA1* diplotypes with clinical outcomes, given the paucity of such data in the literature [15-18, 20, 25, 27, 29, 31, 32], summarized in Supplementary Table 1. Interestingly, despite the fast BU metabolizing capacity and concentrations falling below the target range individuals with rapid metabolizing capacity (*GSTA1 group I*) showed a protective role in terms of OS, likely related to lower probability of TRT. This was most evident in individuals who received BU-cyclophosphamide conditioning regimen. A similar association was seen in a study performed in a Chinese population, where the GSTA1*A*A carriers showed significantly lower AUC than the GSTA1*A*B group with minimal toxicity when BU was administered once daily, highlighting the protective role of GSTA1*A*A [15]. This is also in accordance with our previous report [20] in which patients with two copies of haplotype *A2 had better EFS. On the other hand, individuals in group IV with slow metabolizing capacity demonstrated lower OS. These reductions seem to be related to higher frequency of TRT reflected by more frequent SOS and aGvHD suggesting that these patients are at a higher risk of TRT and might benefit from adjustment of the target AUC and initial dose reduction.

The association of *GSTA1* gene with clinical outcomes might be related to *GSTA1*-PK relationship or might reflect additional involvement of GSTA1 beyond BU metabolism. When both *GSTA1* diplotype groups and BU exposure were entered in a multivariate model, *GSTA1* diplotypes were the only predictors of SOS, whereas aGvHD and combined TRT were predicted by both BU exposure and *GSTA1* diplotypes independently. This was also reflected by the fact that Css above 900 ng/mL was associated with TRT irrespective of diplotype groups, whereas high risk of TRT for Css below 900 ng/mL was evident only for group IV carriers. The patients were recruited during a large time span, which could have influenced some of the associations observed, however, neither prophylactic measures nor TRT incidences differed significantly over time. *GSTA1* also participates in the metabolism of cyclophosphamide [41] possibly explaining modulation of associations of clinical outcomes in BU-cyclophosphamide conditioning regimen. Interestingly in lupus nephritis patients, GSTA1*B genotypes have higher exposure to activated cyclophosphamide metabolites [42]. Therefore, these vulnerable patients might benefit from administration of cyclophosphamide before BU or alternative conditioning regimens such as BU/Fludarabine. Non-catalytic functions of the GST enzymes could be considered as well, for example *GSTM1* and *GSTP1* have been linked to inhibition of the mitogen-activated protein (MAP) kinase pathway [43] modulating apoptotic signalling. Additionally, glutathione S-transferase family has the potential to behave as minor histocompatibility antigens (miHA). miHA disparities have been attributed to GvHD in the HLA-matched transplantation setting [44] potentially explaining independent effect of *GSTA1* and *GSTP1* genotypes in aGvHD susceptibility.

The association observed with *GSTM1* and hemorrhagic cystitis could derive from the interaction between busulfan and cyclophosphamide affecting the clearance of cyclophosphamide metabolites such as acrolein, which can be damaging to the kidney and bladder epithelium [45]. *GSTM1* non-null carriers might deplete the glutathione pool, limiting conjugation of these metabolites by other specific GSTs such as *GSTP1*. The additional role of *GSTM1* in determining clinical outcomes of HSCT, which is distinct from its role in BU metabolism is also a possible explanation for the higher incidences of early mortality observed in the study by Bremer et al. for *GSTM1* non null individuals receiving BU-cyclophosphamide regimen [18].

## CONCLUSION

We provide evidence for an association of *GSTA1* diplotypes with BU PK and clinical outcomes of HSCT supported by functional studies. *GSTA1* diplotypes can explain in some models ∼20% of the variability seen in BU CL and can contribute to HSCT -related complications acting within and beyond BU metabolism. Prior genotyping may be helpful in deciding on BU first dose, thus optimizing therapeutic drug monitoring and decreasing TRT, which is particularly important for *GSTA1 group IV* carriers. It might also help to define each patient risk to toxicity and introduce the possibility of individualised prophylaxis. Other GST polymorphisms seem also to contribute to HSCT-related toxicities and may include additional mechanisms. Further studies are needed to define prospectively how to adjust dose according to the genotype, including non-genetic factors and different BU administration schedules.

## MATERIALS AND METHODS

### Patients

The study includes 138 pediatric patients who underwent allogeneic HSCT with i.v. BU as part of a myeloablative conditioning regimen from five different centers in Europe and Canada (Geneva, Leiden, Montreal, Paris, Toronto), recruited between May 2000 and April 2013. The Institutional Review Board at each center approved the study and all patients and/or parents provided informed consent. Details of inclusion criteria are available at Clinicaltrials.gov site (NCT01257854). Patients’ characteristics are provided in Table 5.

**Table 5:** Participating centers, demographic and transplantation characteristics

Characteristics of the study group			*Patients*	
			*N*	*%*
***Centers***	*CHU St-Justine, Montreal (Canada)*		83	60.1
	*SickKids Hospital, Toronto (Canada)*		25	18.1
	*Geneva University Hospital, Genève (Switzerland)*		4	2.9
	*Robert Debré University Hospital, Paris (France)*		12	8.7
	*University Medical Center, Utrecht (Netherlands)*		14	10.1
***Gender***	*Male*		74	53.6
	*Female*		64	46.4
***Ethnicity (n = 135)***	*Caucasian*		99	73.3
	*Native American*		4	3
	*African-American*		20	14.8
	*Asian*		9	6.7
	*Middle East*		3	2.2
***Diagnosis***				
*Malignancies*	*ALL/AML*		43	31.2
	*MDS*		31	22.5
	*ALL*		12	8.7
	*Biphenotypic acute leukemia*		1	0.7
	*Myeloproliferative syndrome*		1	0.7
*Non-Malignancies*	*Hemoglobinopathy*		14	10.1
	*Immunodeficiencies*		13	9.4
	*Hemophagocytic syndrome*		12	8.7
	*Metabolic disease*		8	5.8
	*BMFS*		3	2.2
***HLA compatibility***	*MUD*		30	21.7
	*MM – related donor*		6	4.3
	*MM – unrelated donor*		53	38.4
	*HLA identical sibling*		49	35.5
***Stem Cell Source***	*BM*		59	42.8
	*PBSC*		4	2.9
	*Cord Blood*		74	53.6
	*BM + PBSC*		1	0.7
***Myeloablative Conditioning***	*BU/CY*		106	76.8
	*BU/Mel*		2	1.4
	*BU/CY/Mel*		15	10.9
	*BU/CY/VP16*		15	10.9
***Serotherapy***	*No*		37	26.8
	*ATG*		99	71.7
	*Campath*		2	1.4
***GvHD Prophylaxis***	*CSA + steroids*		50	44.64
	*CSA + MTX*		51	45.53
	*CSA*		9	8.03
	*No*		2	1.8
	***Median***	***Range***		
***BM:***				
*Nucleated cells (x10*^*8*^*/Kg)/n=57)*	4.91	(0.11-23.2)		
*CD34 cells (x10*^*8*^*/Kg)/n=39)*	0.05	(0.00024-1.3)		
***Cord Blood:***				
*Nucleated cells (x10*^*8*^*/Kg)/n=74)*	1.2	(0.7-14.8)		
*CD34 cells (x10*^*8*^*/Kg)/n=73)*	0.0035	(0.0001-0.14)		
***PBSC:***				
*Nucleated cells (x10*^*8*^*/Kg)/n=4)*	3.49	(0.01-47.1)		
*CD34 cells (x10*^*8*^*/Kg)/n=4)*	0.17	(0.014-0.31)		
*Age (years)*	5.8	(0.1-19.9)		
*Weight (kg)*	20.05	(4-87.9)		
*Height (cm)*	112.5	(51-183)		
*BMI (Kg/m*^*2*^*)*	17.43	(12.9-29.5)		
*BSA (m*^*2*^*)*	0.8	(0.24-2.1)		

Abbreviations ALL: acute lymphoblastic leukemia; AML: acute myeloid leukemia; ATG: anti-thymocyte globulin; BM: bone marrow; BMFS: bone marrow failure syndrome; BMI: Body mass index; BSA: body surface area; BU: busulfan; Campath: Alemtuzumab; CY: cyclophosphamide; CSA: cyclosporine; Flu: fludarabine; MDS: myelodysplastic syndrome; Mel: melphalan; MM: mismatch; MRD: matched related donor; MUD: matched unrelated donor; MTX: methotrexate; CSA: cyclosporine; SD: sibling donor; GvHD: graft-versus-host-disease; PBSC: peripheral blood stem cells; VP16:etoposide

### Sampling, genotyping, administration of BU and PK estimation

GST genotyping was performed according to the previously described procedures [33, 46]. BU was administered every 6h as a 2 h infusion for a total of 16 doses. The first dose was either age or weight-based and dose adjustment (based on the first dose PK parameter estimates) was performed on the 3^rd^; 4^th^; 5^th^; or 9^th^ dose onwards as detailed on Table 6 which summarizes the dosing schedule followed by each center. The median BU PK parameters observed in the study subjects after administration of the first dose are summarized in Table 7.

**Table 6:** Administration of BU and Pharmacokinetic parameter estimation per Center (n=138)

Centers	Busulfan Initial IV Dose	Busulfan, Dose adjusted	Method for estimating Busulfan concentration	Therapeutic Drug Target	The determination of pharmacokinetic parameters, estimated from the first dose.
**CHU St-Justine, Canada**	0.8mg/kg/dose (infants≥3months and <1 year of age) 1 mg/kg/dose (children≥1 year and <4 years old) 0.8mg/kg/dose (children≥4 years old)	5th dose	HPLC/LC-MS/MS	Css target = 600–900 ng/mL	Non-compartmental analysis (WinNonlin, version 3.1, Pharsight)
**Geneva University Hospital, Switzerland**	0.8mg/kg/dose (infants≥3months and <1 year of age) 1 mg/kg/dose (children≥1 year and <4 years old) 0.8mg/kg/dose (children≥4 years old)	4th dose	LC-MS/MS	Css target = 600–900 ng/mL	Non-compartmental analysis (WinNonlin, version 3.1, Pharsight)
**Leiden University Medical Center Netherlands**	0.8 to 1mg/kg/dose (infants≥4 years of age); 1mg/kg/dose (infants<4 years old)	5th or 9th dose	HPLC/LC-MS/MS	Css target = 740–910 ng/mL Then the dose was only adjusted by a maximum of 1 mg/kg every 6 hours.	Non-compartmental analysis (WinNonlin, version 3.1, Pharsight)
**Robert Debré University Hospital, France**	<9kg (1mg/kg/dose) 9≥to<16kg (1.2mg/kg/dose) 16≥to≤23kg (1.1mg/kg/dose) >23to≤34kg (0.95mg/kg/dose) >34kg (0.8mg/kg/dose)	7th or 9th dose	GC-MS	Css target = 600-1026 ng/mL	Non-compartmental analysis (WinNonlin, version 3.1, Pharsight)
**SickKids Hospital, Canada**	<9kg (1mg/kg/dose) 9 to <16kg (1.2mg/kg/dose) 16 to 23kg (1.1mg/kg/dose) 23 to 34kg (0.95mg/kg/dose) >34kg (0.8mg/kg/dose)	3rd or 4th dose	GC-ECD	Css target = 889 ng/mL	Limited Sampling Strategy or Trapezoidal rule to calculate AUC

Evaluation of Pharmacokinetic parameter estimations: A cross calibration study was conducted by our group with the collaboration of Pierre Fabre Laboratories between all centers participating in the study to validate analytical method used to determine the PK parameters from the first dose (data available upon request). Only centers with the measured BU concentrations falling within ± 20% of the therapeutic drug target concentrations were included for analysis of PK parameters. Abbreviations: AUC: Area under the curve; Css: Steady state concentration; GC-MS: Gas Chromatography Mass Spectrometry; GC-ECD: Gas Chromatography with Electron Capture Detector; HPLC: High Performance Liquid Chromatography; LC-MS/MS:Liquid chromatography–mass spectrometry

**Table 7:** Busulfan pharmacokinetic parameters observed in the study subjects after administration of the first dose.

Parameter for all centers	Median (range) N=138	Median (range) CHUSJ only N=83	Median (range) Other cohorts N=55
C_max_ (ng/mL)	890.5 (515.9-1709)	844.0 (545.0-1298.0)	1004.8 (515.9-1709)
C_SS_ (ng/mL)	667.7 (325-1238)	596.0 (325.0-1227.0)	711.0 (403.0-1238.0)
AUC (mg.h/L)	3.5 (1.82-7.31)	3.3 (1.8-7.2)	4.2 (2.2-7.3)
Clearance (mL/min/kg)	4.1 (1.8-7.2)	4.2 (1.8-7.2)	3.7 (1.8-6.3)

Cmax: maximum plasma concentration; Cmin: Minimum plasma concentration; Css: Steady state plasma concentration; AUC: Area under the curve; CHUSJ: Center Hospitalier universitaire Sainte-Justine

Clinical outcomes (Table 8) were defined as per the standard guidelines of the European Society for Blood and Marrow Transplantation and Center for International Blood and Marrow Transplant Research as detailed in previous reports [6, 20]. TRT was defined as the occurrence of first toxicity, either SOS, aGvHD, lung toxicity or HC. An event was defined as graft rejection, relapse or death from any cause.

**Table 8:** Clinical outcomes observed in the study subjects

Clinical outcomes		Cumulative incidence		Day of onset	
		**N**	**(%)**	**Median**	**(range)**
Neutrophil recovery (Day 100)		123	(89.1)	19	(1-50)
Platelet recovery (Day 180)		106	(77)	40	(16-173)
Sinusoidal Obstruction Syndrome (SOS)		14	(10.1)	16	(3-47)
aGvHD (grade 1-4)		43	(31.2)	36	(10-162)
aGvHD (grade 2-4)		27	(19.6)	44	(11-162)
Lung toxicity		7	(5.1)	45	(4-166)
Hemorrhagic cystitis		26	(18.8)	24.5	(2-113)
Death		38	(27.5)	127	(15-365)
Combined Treatment Related Toxicity (TRT)		55	(39.8)	136	(2-180)
Rejection		12	(8.7)	52.5	(34-246)
Event		45	(33)	70	(15-364)
% of donor cell chimerism Day 100 (n=112)	>95% ≥50%-95% <50%	66 13 19	(58.9) (11.6) (17)	96 100 95	(24-132) (31-118) (28-156)

Abbreviation: aGvHD - acute Graft versus Host Disease

### Reporter-gene assay

Plasmid constructs were prepared by a gene assembly service (GeneScript, Piscataway, USA). DNA region from -1275 to +126 relative to the translational start codon was cloned into pGl4.10 (Promega, Maddison, USA). HepG2 cells were co-transfected with the pGL4.10 *GSTA1* constructs (with site specific mutations underlying *GSTA1* haplotypes) and the pRL-SV40 vector (Promega) that codes for *Renilla* luciferase for transfection control and normalisation. Difference in promoter activity between haplotypes was assessed by t-test or ANOVA.

### Statistical analyses

BU clearance (mL/min/kg), ratio of adjusted to initial dose and cumulative AUCs were compared across genotypes or diplotype groups using non-parametric tests or linear regression. Cumulative incidences of OS and EFS and of adverse events were estimated in relation to the genotype/ diplotype groups, using Kaplan-Meier framework and log-rank test. Univariate Cox regression was used to estimate hazard ratios. The analyses were also performed by cumulative incidence of competing events and the difference among groups estimated by Gray’s test [47]. The relationship of *GST* with CL and clinical outcomes was additionally explored through stratified and multivariate analysis. Stratified analyses were performed according to age, gender, diagnosis and conditioning regimen. Multivariate analysis included co-variables that were either significantly associated with outcome studied, correlated with genotypes/diplotypes or modulated genotype-phenotype associations: age, gender, diagnosis, conditioning regimen, ethnicity and BU exposure. The allele and genotype frequencies, and Hardy-Weinberg equilibrium were analysed using Haploview [48]; haplotypes were resolved using PHASE [49]. Statistical analyses were performed using IBM^®^ SPSS^®^ statistics (version 19, SPSS Inc, NY) and EZR (Version 1.31) [50].

## SUPPLEMENTARY MATERIALS TABLE





## Abbreviations

HSCT: Hematopoietic stem cell transplantation
GST: glutathione S transferase
GSTA1: glutathione S transferase Alpha1
GSTP1: glutathione S transferase Pi 1
GSTM1: glutathione S transferase Mu 1
GSTT: glutathione S transferase Theta
BU: Busulfan
AUC: Area under the curve
Css: Stead state concentration
PK: pharmacokinetics
TRT: Treatment Related Toxicity
SOS: Sinusoidal Obstruction Syndrome
aGvHD: acute Graft versus Host Disease
HepG2: Human hepatoma
OS: Overall survival
EFS: Events Free Survival
