# Supplementary material for: GSTA1 diplotypes affect busulfan clearance and toxicity in children undergoing allogeneic hematopoietic stem cell transplantation: a multicenter study
Source: Oncotarget. 2017 Aug 27;8(53):90852–67. doi: 10.18632/oncotarget.20310 (PMC5710889; doi:10.18632/oncotarget.20310)
Supplement: Supplementary file 1 [file oncotarget-08-90852-s001.pdf]

GSTA1 diplotypes affect busulfan clearance and toxicity in children undergoing allogeneic hematopoietic stem cell transplantation: a multicenter study

Supplementary Materials

For Supplementary Table see in Supplementary Files
