# Supplementary material for: GSTA1 diplotypes affect busulfan clearance and toxicity in children undergoing allogeneic hematopoietic stem cell transplantation: a multicenter study
Source: Oncotarget. 2017 Aug 27;8(53):90852–67. doi: 10.18632/oncotarget.20310 (PMC5710889; doi:10.18632/oncotarget.20310)
Supplement: Supplementary file 2 [file oncotarget-08-90852-s002.docx]

|  | **Supplementary Table 1.** Pharmacogenetic association studies of GST genes with busulfan pharmacokinetics and HSCT clinical outcomes | | | | | | |
| --- | --- | --- | --- | --- | --- | --- | --- |
| **Regimen** | **BU**  **formulation/dosing** | **BU Dose adjust-**  **ment** | **Genes** | ***GSTA1* Association with Pharmacokinetics of BU** | ***GSTs* Association with clinical outcomes and toxicity** | **Indication for HSCT, Patient population (n)** | **Reference** |
| BU-CY | i.v./QD | No | *GSTA1* and  *P1* | Decreased CL by 28% in *GSTA1*A*B* carriers | No significant association | Malignancies, Adult, Asian (25) | Yin et al[[15](#_ENREF_15)] |
| BU-CY or BU-Flu | i.v./QID & QD | No | *GSTA1,M1,T1,* and *P1* | Decreased CL by 15% in *GSTA1*A*B* carriers | No significant association | Malignancies and Non-malignancies, Adult, Asian (36) | Choi et al[[16](#_ENREF_16)] |
| BU-CY | i.v./QID | Yes | *GSTA1, M1* | Decreased CL by 7%, 50% in *GSTA1*A*B & *B*B* carriers, respectively | Association of  *GSTM1 null* genotype and regimen related toxicity was observed | Hemoglobinopathies , Pediatric, Middle-East (44) | Ansari et al[[17](#_ENREF_17)] |
| BU-CY | Oral/QID | Yes | *GSTA1, M1, T1,*  and *P1* | Decreased CL/F by 18% in *GSTA1*B*B* carriers | No direct significant association | Malignancies, Adult, Caucasian (114) | Bremer et al[[18](#_ENREF_18)] |
| BU-CY- Hu-Az-Flu/BU-CY-Flu | i.v./QID | Yes | *GSTA1, M1, T1,*  and *P1* | No significant association | No significant association | Hemoglobinopathies, Pediatric African,Caucasian (36) | Gaziev et al[[29](#_ENREF_29)] |
| BU-CY +/- Mel or Eto & BU-Flu+/-Mel or Thio or Clo | i.v./QD | Yes | *GSTA1, CYP2C19, CYP39A1, ABCB4, SLC22A4 & SLC7A8* | Decreased CL by 26% in *GSTA1*B *B* carriers | Not investigated | Malignancies and Non-malignancies Pediatric, Caucasian (84) | Ten Brink et al[[19](#_ENREF_19)] |
| BU-Flu/BU-CY-Flu | i.v./QD | No | 1936 SNPs in ADME genes | Decreased CL by 18% in *GSTA1*B*B* carriers (inferred as it is in linkage with SNPs in *GSTA5* reported in the study) | Not investigated | Malignancies and Non-malignancies Adult, Caucasian (62exploration, 78 validation) | Ten Brink et al[[30](#_ENREF_30)] |
| BU-CY or Mel | i.v./QID | Yes | *GSTA1,M1,T1* and *P1* | Increased CL by 22% in *GSTA1 *A2*A2* carriers | *GSTA1*A2* with better EFS *GSTA1*B* with higher risk of SOS and *GSTM1-null* with acute GVHD | Malignancies and Non-malignancies, Pediatric, Caucasian (69) | Ansari et al[[20](#_ENREF_20)] |
| BU-CY/BU-Flu | i.v./QID | No | *GSTA1, M1, T1,*  and *P1* | Decreased CL by 29% in *GSTA1*B*B* carriers | No significant association | Malignancies and Non-malignancies, Adults, Caucasian (66) | Ten Brink et al[[21](#_ENREF_21)] |
| BU-CY | i.v./QID&QD | No | *GSTA1,M1,* and *T1* | Decreased CL by 12% in *GSTA1*A*B* carriers | Not investigated | Malignancies, Adult, Asian (60) | Kim et al[[22](#_ENREF_22)] |
| BU-CY/BU-Flu/BU-Flu-CY/BU-Mel | i.v. and oral/QID | Yes | *GSTA1* and *M1* | Decreased CL/F by 16% in *GSTA1*B*B* carriers. No association in case of i.v. BU CL. | Not investigated | Malignancies and Non-malignancies Adult and pediatric Caucasian mainly (152) | Abbasi et al[[23](#_ENREF_23)] |
| BU-CY- Hu-Az- /BU-CY+/-Thio | i.v./QID | Yes | *GSTA1,M1,T1* and *P1* | Decreased CL by 10% in *GSTA1*B* carriers | Not investigated | Hemoglobinopathies, Pediatric, Caucasian (71) | Gaziev et al[[24](#_ENREF_24)] |
| BU-CY | Oral/QID | Yes | *GSTA1,M1* and *P1* | Decreased CL by 5% in *GSTA1*B*B* carriers | Association between aGvHD  and *GSTM1*-null genotype was observed | Hemoglobinopathies , Pediatric, Arab (18) | Elhasid et al[[25](#_ENREF_25)] |
| BU-CY or Flu | i.v./QID&BID | Yes | *GSTA1,M1* and *P1* | Decreased CL by 30% in *GSTA1*B* carriers | Not investigated | Malignancies and Non-malignancies, Pediatric, Caucasian mainly (27) | Johnson et al[[26](#_ENREF_26)] |
| BU-CY | Oral/NS | No | *GSTA1* | Not investigated | GvHD incidence lower in individuals with two *GSTA1*A* copies | Malignant, Adult, Asian (61) | Kim et al[[27](#_ENREF_27)] |
| BU-CY or Flu or Mel or etoposide combinations | i.v./QID&QD | yes | *GSTA1,M1,T1* and *P1* | No association with population CL | *GSTM1-null* with SOS (trend) | Malignancies and Non-malignancies, Pediatric, Caucasian (77) | Zwaveling et al. [[31](#_ENREF_31)] |
| BU-CY | Oral/QID | No | *GSTA1* | Decreased CL/F by 40% in *GSTA1*A*B* carriers | Not investigated | Malignant, Adult, Asian (12) | Kusama et al[[28](#_ENREF_28)] |
| BU-CY | Oral/QID | No | *GSTM1,T1* | Higher CL and lower Css in *GSTM1- null* individuals | Higher SOS occurrence in *GSTM1 -null* individuals | Hemoglobinopathies , Pediatric, Asian (114) | Srivastava et al.[ [32](#_ENREF_32)] |

*Patients with malignancies (lymphoid, myeloid hematological cancers, myelodysplastic syndrome and myeloproliferative disease). Non-malignant cases (hemoglobinopathies, immunodeficiency, metabolic and, granulomatous disease, hemoglobinopathies). *A and *B haplotypes in GSTA1 are defined by -69C>T. ADME: absorption, distribution, metabolism and elimination. ; Az: azathioprine ; BU: busulfan; BID: Twice Daily; CL: Clearance, Css: Steady state concentration; Clo:clofarabine; CY: cyclophosphamide; Eto: Etoposide; Flu: fludarabine; F:Bioavailability; Hu: hydroxyurea; Mel: melphalan; QD: Once Daily also known as Q24H; Dosing; QID: Four times daily dosing also called as Q6H; Thio:Thiotepa.* Please refer to the main text for references cited in this table.
